# Supplementary material for: AN1-type zinc finger protein 3 (ZFAND3) is a transcriptional regulator that drives Glioblastoma invasion
Source: Nat Commun. 2020 Dec 11;11:6366. doi: 10.1038/s41467-020-20029-y (PMC7732990; doi:10.1038/s41467-020-20029-y)
Supplement: Supplementary file 1 — Supplementary Information [file 41467_2020_20029_MOESM1_ESM.pdf]

Supplementary files - Schuster et al.

AN1-type zinc finger protein 3 (ZFAND3) is a transcriptional regulator that drives Glioblastoma invasion

Supplementary Figures

Supplementary Figure 1

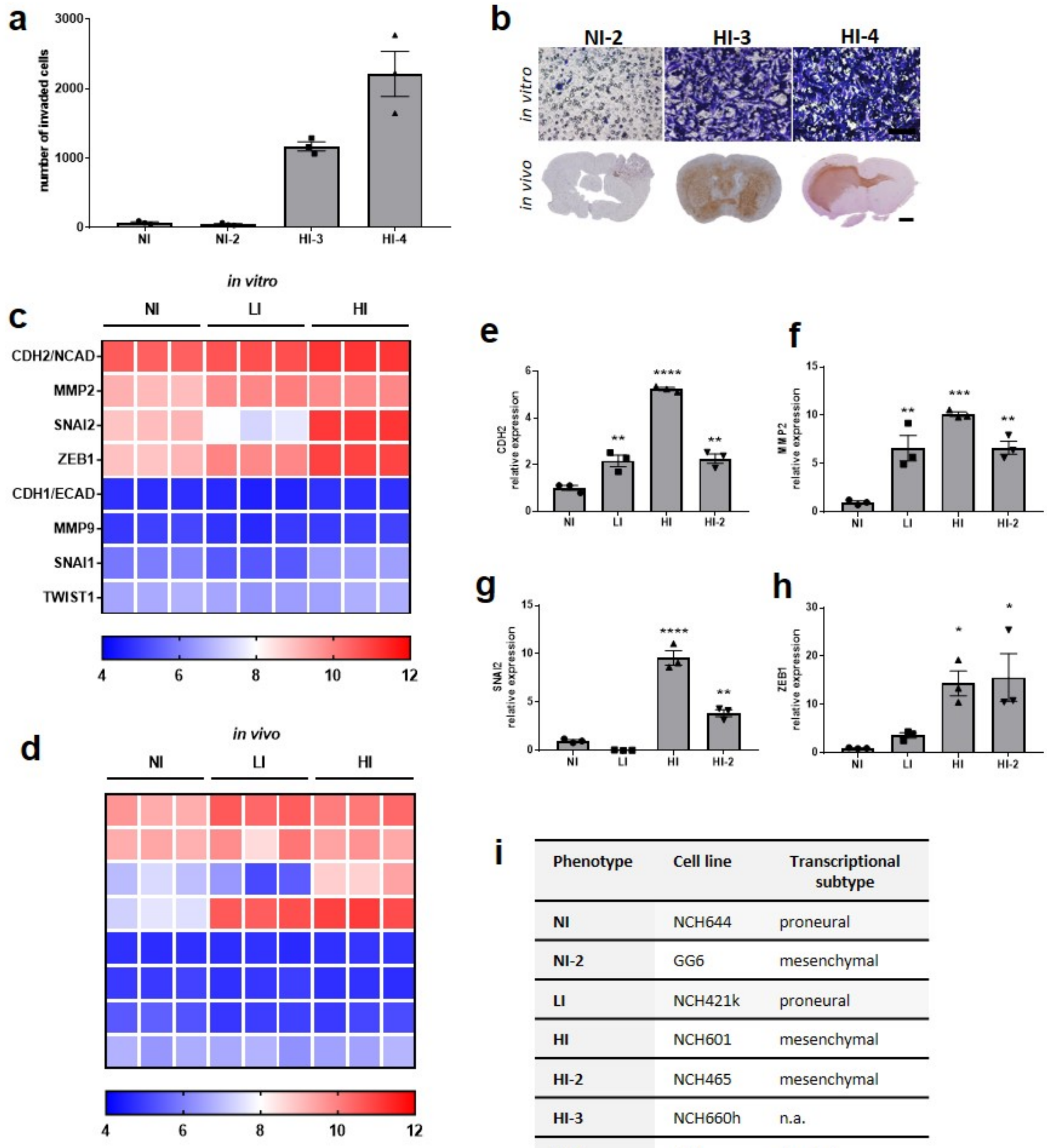

Phenotype	Cell line	Transcriptional subtype
**NI**	NCH644	proneural
**NI-2**	GG6	mesenchymal
**LI**	NCH421k	proneural
**HI**	NCH601	mesenchymal
**HI-2**	NCH465	mesenchymal
**HI-3**	NCH660h	n.a.
**HI-4**	BG7	classical

Supplementary Figure 1: Characterization of patient-derived GSC lines. *a-b) Invasion capacities of additional GSC lines:* (a) Quantification of *in vitro* invasion in Boyden chamber assays (n=3 biologically independent experiments) of 2 non-invasive (NI, NI-2) and two highly invasive (HI-3, HI-4) GSC lines. (NI line is shown as comparison to Fig. 1). Results are displayed as average number of invaded cells +/- SEM. (b) Representative pictures of *in vitro* invasion (Boyden chamber assays) and *in vivo* invasion in orthotopic mouse xenografts (except NI-2 in rat). Scale bar = 1000 µm. c-h) The expression of general invasion markers reflects the status of non invasive (NI), invasive (LI), highly invasive (HI, HI-2) GSCs. Genome-wide expression analysis revealed differential expression of *CDH2*, *MMP2*, *SNAI2*, *ZEB1*, but absence of expression of *CDH1*, *MMP9, SNAI1, TWIST1* in GSCs *in vitro* (c) and at endpoint of tumor development (d) in orthotopic xenografts *in vivo*. qPCR showing expression of *CDH2* (n=3) (e), *MMP2* (n=3) (f), *SNAI2* (n=3) (g) and *ZEB1* (n=3) (h) in different types of GSCs. Results are displayed as average +/- SEM and were anlaysed with an ordinary one-way ANOVA *$p_{value}<0.05$; **$p_{value}<0.01$; ***$p_{value}<0.001$; ****$p_{value}<0.0001$. i) List of human GSC lines indicating their invasive phenotype (left panel) and transcriptional GBM subtype (right panel) based on gene expression and/or methylation profiling.

Supplementary Figure 2

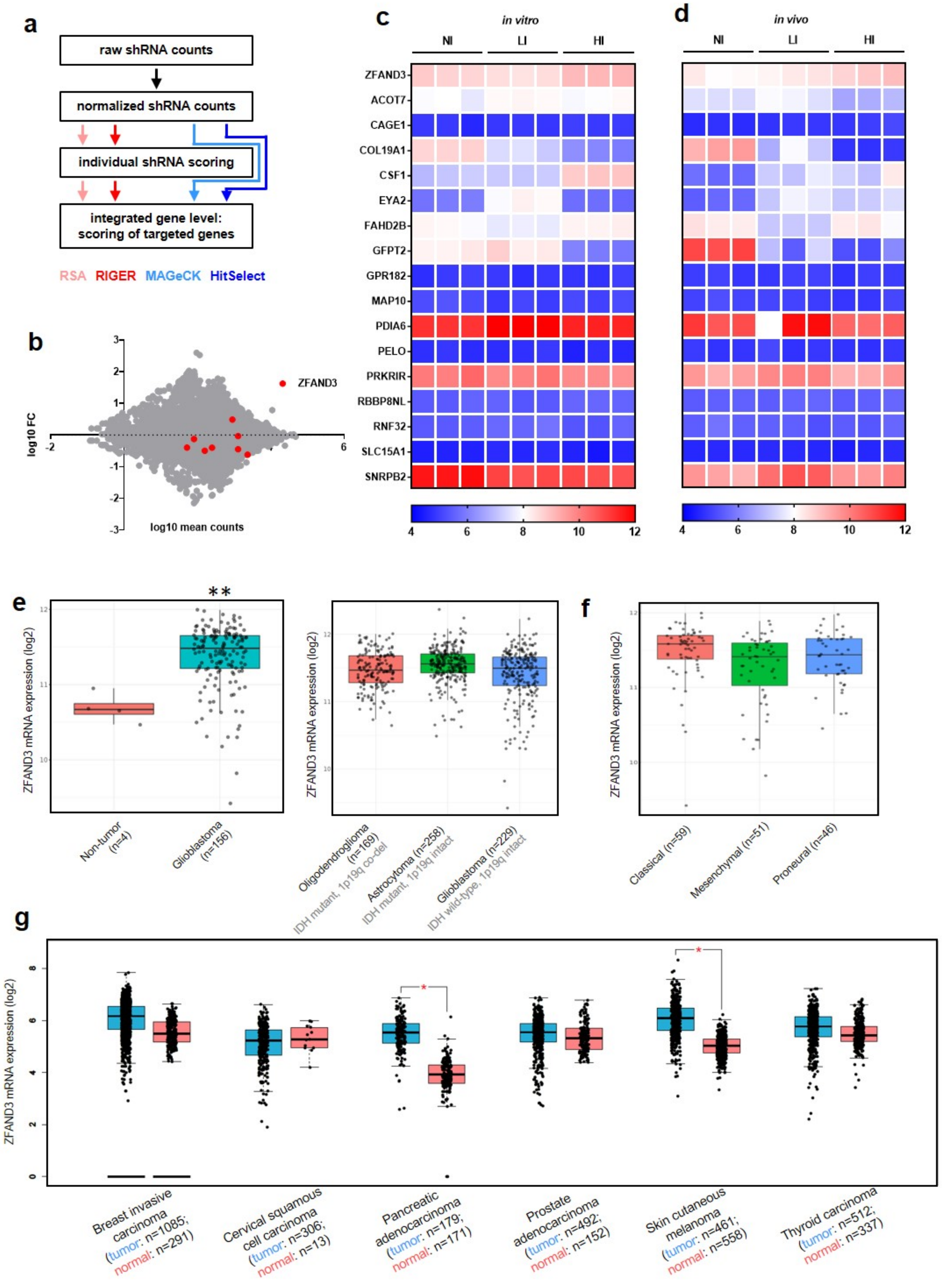

Supplementary Figure 2: Genome-wide shRNA analysis identifying ZFAND3 as top candidate. a) Analysis pipeline of genome-wide shRNA interference screen. b) shRNA distribution based on RSA analysis shows that shRNAs targeting *ZFAND3* appear enriched (7 out of 8) in invasion-defective cells (log10FC<0). Genome-wide expression analysis shows the expression of the 17 genes identified by the shRNA screen in different types of GSCs c) *in vitro* and d) *in vivo* at endpoint of tumor development in orthotopic xenografts. e) Analysis of *ZFAND3* expression in patients from TCGA database, via the GlioVis platform (GBM and GBM/LGG cohort, RNAseq data) [1]. Data were analysed with Tukey's Honest Significance Difference, after one-way ANOVA. ** $p_{value}<0.01$ f) *ZFAND3* expression in GBM patients from TCGA database according to GBM transcriptional subtype. Whiskers of dot plots represent extreme low and high values as long as they are within the 1.5 times interquartile range (IQR), meaning that whiskers are limited to 1.5 IQR. Dots above and below whiskers display outlying data points. Centres represent the medians and the box limits show the 25th and 75th percentiles. g) *ZFAND3* expression among different cancer types (tumor in blue, normal tissue in red). Data collected from GEPIA [2] based on TCGA normal and GTEx data. Data were analysed with one-way ANOVA, using disease state as variable for calculating differential expression. * $p_{value}<0.05$. The boxes of the box plot display data from the 25th to 75th percentile, while the whiskers show the 10th and 90th percentile. The centres represent the medians of the data.

Supplementary Figure 3

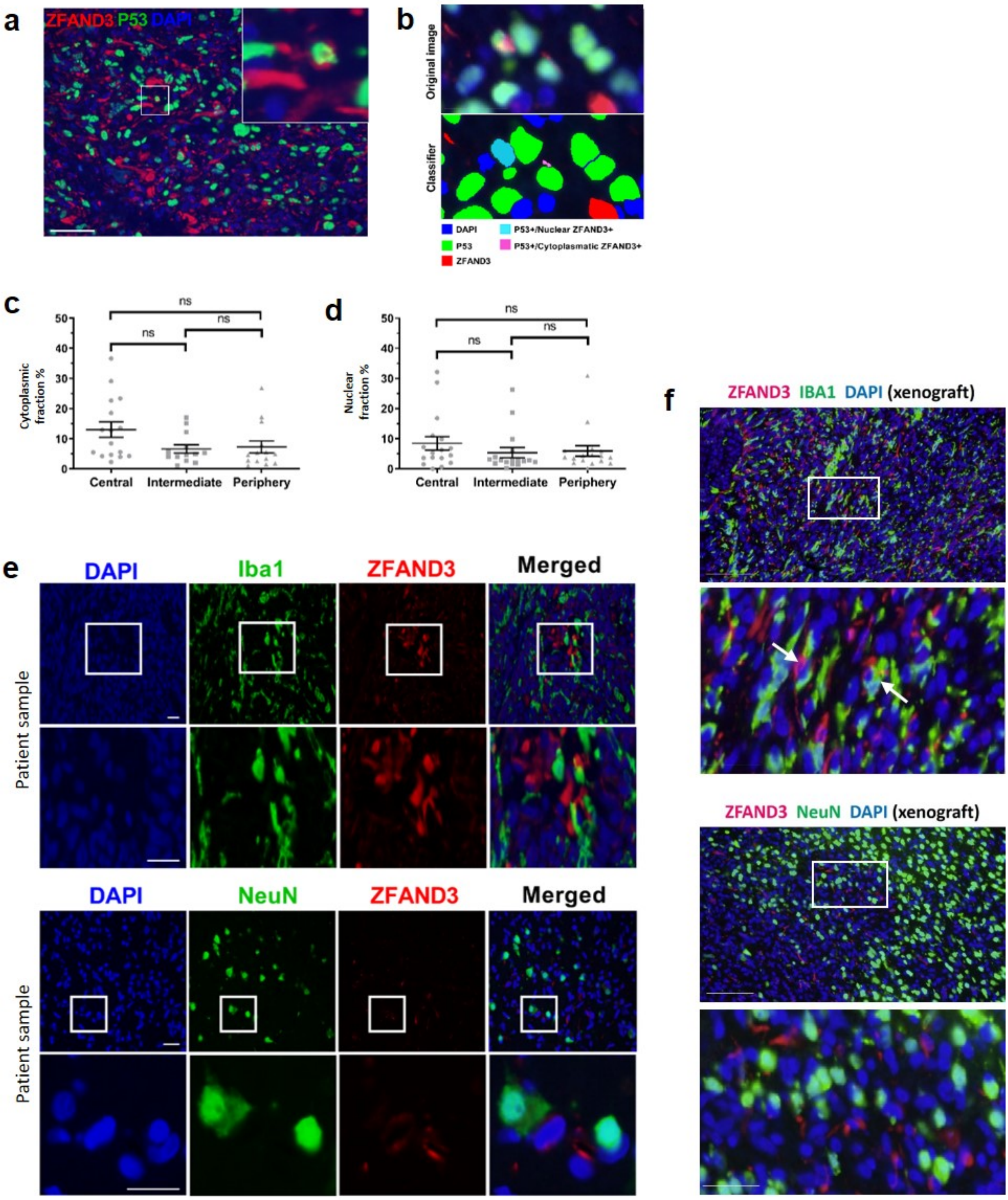

Supplementary Figure 3: ZFAND3 protein expression in GBM patient samples and xenografts. a) Representative picture of P53/ZFAND3 double-fluorescence stainings in GBM patients. (Scale bar = 50µm). (n=17 patient samples) b) Representative picture of a central tumor region with and without the application of a software based cell-classifier. Identification of DAPI-positive nuclei (blue), P53-positive nuclei (green),

ZFAND3 staining (red), cells with P53+/ZFAND3+ cytoplasmic expression (pink) and cells with P53-/ZFAND3+ nucelar expression (turquoise). Scale bar = 10 µm c-d) Quantifcation of tumor cell fraction with cytoplasmic (c) or nuclear (n=17 patient samples) (d) ZFAND3 positivity on three tumor regions (central, intermediate, peripheral). e) Double-immunofluoresence of ZFAND3 with Iba1 (upper panel) or NeuN (lower panel) in GBM patients (Scale bar upper rows = 25 µm, lower rows = 15 µm). (n=9 different patient samples) f) Double-immunofluoresence of ZFAND3 with Iba1 (upper panel) or NeuN (lower panel) in HI GSC xenograft. Scale bar = 100 µm on overview images and 25 µm on magnified images(n=4 mice). For c and d resuts are displayed as average +/ SEM and data were analysed as matched data with one-way ANOVA and Tukey's multiple comparison test,

Supplementary Figure 4

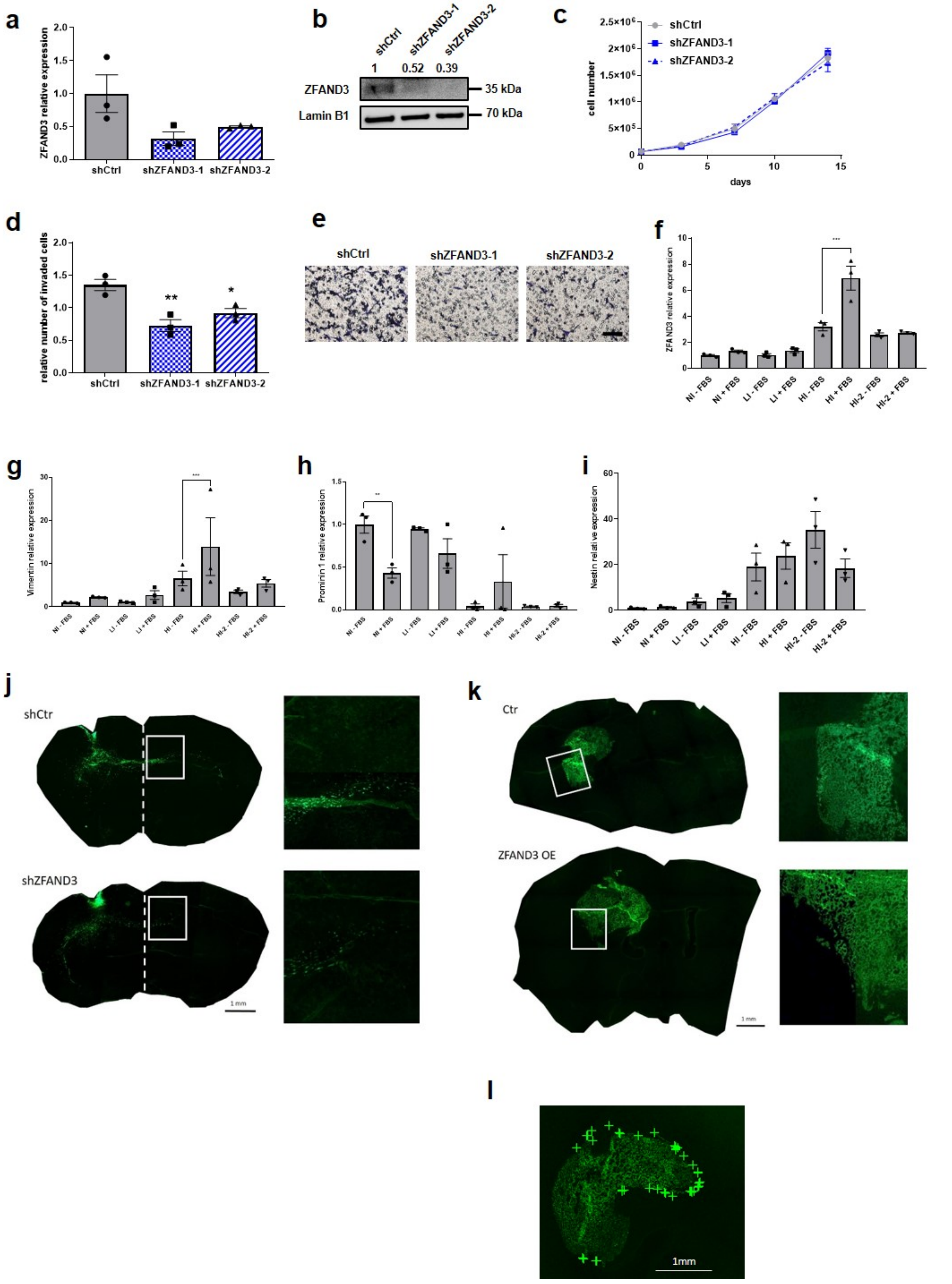

Supplementary Figure 4: ZFAND3 knockdow in 2nd HI cells, gene expression during invasion assay and overview images of xenografts. a) qPCR showing *ZFAND3* expression upon knockdown (KD) in highly invasive GBM cells (HI-2) (n=3 biologically independent samples). Results are displayed as average +/- SEM and were anlaysed with an ordinary one-way ANOVA b) Western-blot showing protein expression upon ZFAND3 KD (n=3). c) Growth curves of control and ZFAND3 KD cells (n=3). d) *In vitro* Boyden chamber invasion assays of control and ZFAND3 KD cells (n=3 biologically independent experiments). Results are displayed as average +/- SEM and were analysed with an ordinary one-way ANOVA.e) Representative pictures of *in vitro* Boyden chamber invasion assays. Scale bar = 100 µm. f) *ZFAND3* expression in different GSC models under *in vitro* invasion conditions (7% FBS in medium) (n=3). Results are displayed as average fold change +/- SEM and were analysed with an ordinary one-way ANOVA. g-i) Expression of stem cell markers in different GSC models under *in vitro* invasion conditions (n=3). Results are displayed as average fold change +/- SEM and were analysed with an ordinary one-way ANOVA. j) Overview image of HI GSC xenograft with control (shCtr) and ZFAND3 knockdown (shZFAND3). GFP positive tumor cells invading to the contralateral brain hemisphere were counted (boxed area and blow up). k) Overview image of NI GSC xenograft of control (ctr) or ZFAND3 overexpressing cells. GFP positive tumor cells at the edge of the tumor area were counted as shown in (l). *$p_{value}<0.05$; **$p_{value}<0.01$; ***$p_{value}<0.001$.

Supplementary Figure 5

a

MGDAGSERSKAPSLPPRCPCGFWGSSKTMNLCSKCFADFQKKQPDDDSAPSTSNSQSDLFSEETTSDNNNTSITTP
TLSPSQQPLPTELNVTSPSKEECGPCTDTAHVSLITPTKRSCGTDSQSENEASPVKRPRLLENTERSEETSRSKQK
SRRRCFQCQTKLELVQQELGSCRCGYVFCMLHRLPEQHDCTFDHMGRGREEAIMKMVKLDRKVGRSCQRIGEGCS

NLS site, AAs mutated to Alanine in ZFAND3mutNLS in bold/underlined (K132A, R133A and R135A)

Zinc finger_A20-type (AA 15-44) deleted in ZFAND3-Δ1

Zinc Finger_AN1-type (AA 155-198) deleted in ZFAND3-Δ2

AA: Zinc complexing amino acids. AAs mutated to Alanine are underlined (C32A + C35A in ZFAND3 M1; C176A +C181A in ZFAND3 M2; all four in ZFAND3 M1-M2)

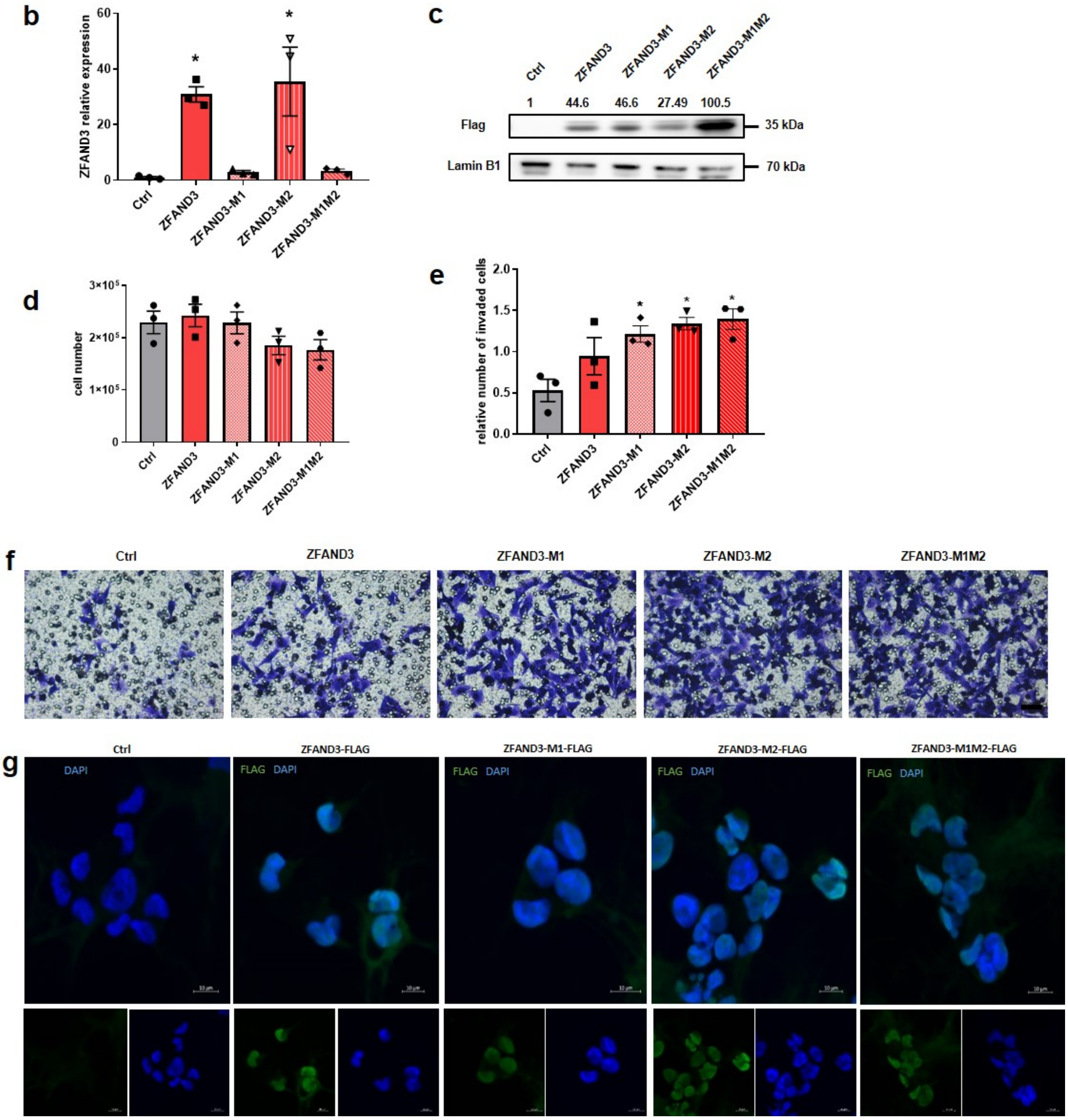

Supplementary Figure 5: ZFAND3 mutant constructs. a) ZFAND3 protein sequence indicating domain areas and mutant constructs used in this manuscript. b) Validation of expression of ZFAND3 wildtype and point mutation constructs (M1, M2, M1-M2) in NI GSCs by qPCR (n=3 biologically independent samples) (b) and Western blot (n=2) (c). Note that for M1, primers in qPCR did not bind efficiently because of overlapping point mutations. d) No effect on proliferation was seen with respective constructs (day 3, n=3 biologically independent experiments). e) *In vitro* Boyden chamber invasion assays of control cells and cells harboring mutations in the ZFAND3 zinc finger domains (n=3 biologically independent experiments). (f) Representative pictures of *in vitro* Boyden chamber invasion assays. Scale bar = 100 µm. (g) Nuclear localization of ZFAND3 in cells with mutations in zinc finger domains was maintained (n=3). (green: FLAG, blue: DAPI. Scale bars = 10 µm). Results are displayed as average +/- SEM and for b) and e) results were analysed with an ordinary one-way ANOVA. *p_{value}<0.05.

Supplementary Figure 6

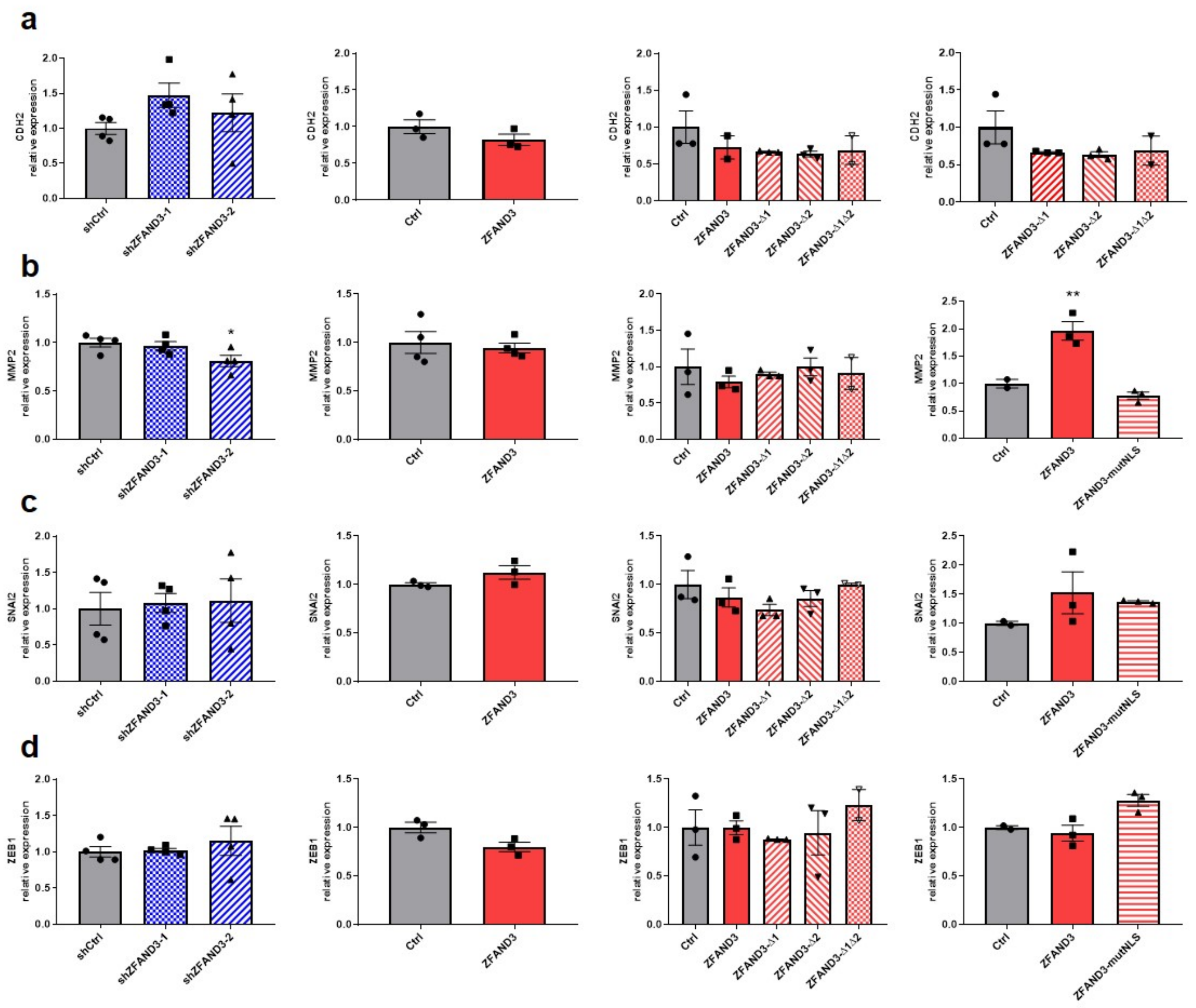

Supplementary Figure 6: qPCRs showing gene expression of general invasion markers upon ZFAND3 knockdown, overexpression, zinc-finger deletions and mutation in nuclear localization signal. a-d) qPCR showing expression of invasion-related genes (*CDH2, MMP-2, SNAI2, ZEB1*) in NI GSCs upon modulation of ZFAND3 expression by knockdown (blue) or overexpression of either wildtype ZFAND3 or mutant constructs (red). Results are displayed as average +/- SEM and were analysed with an ordinary one-way ANOVA (n= 2-4). **p_{value}<0.01.

Supplementary Figure 7

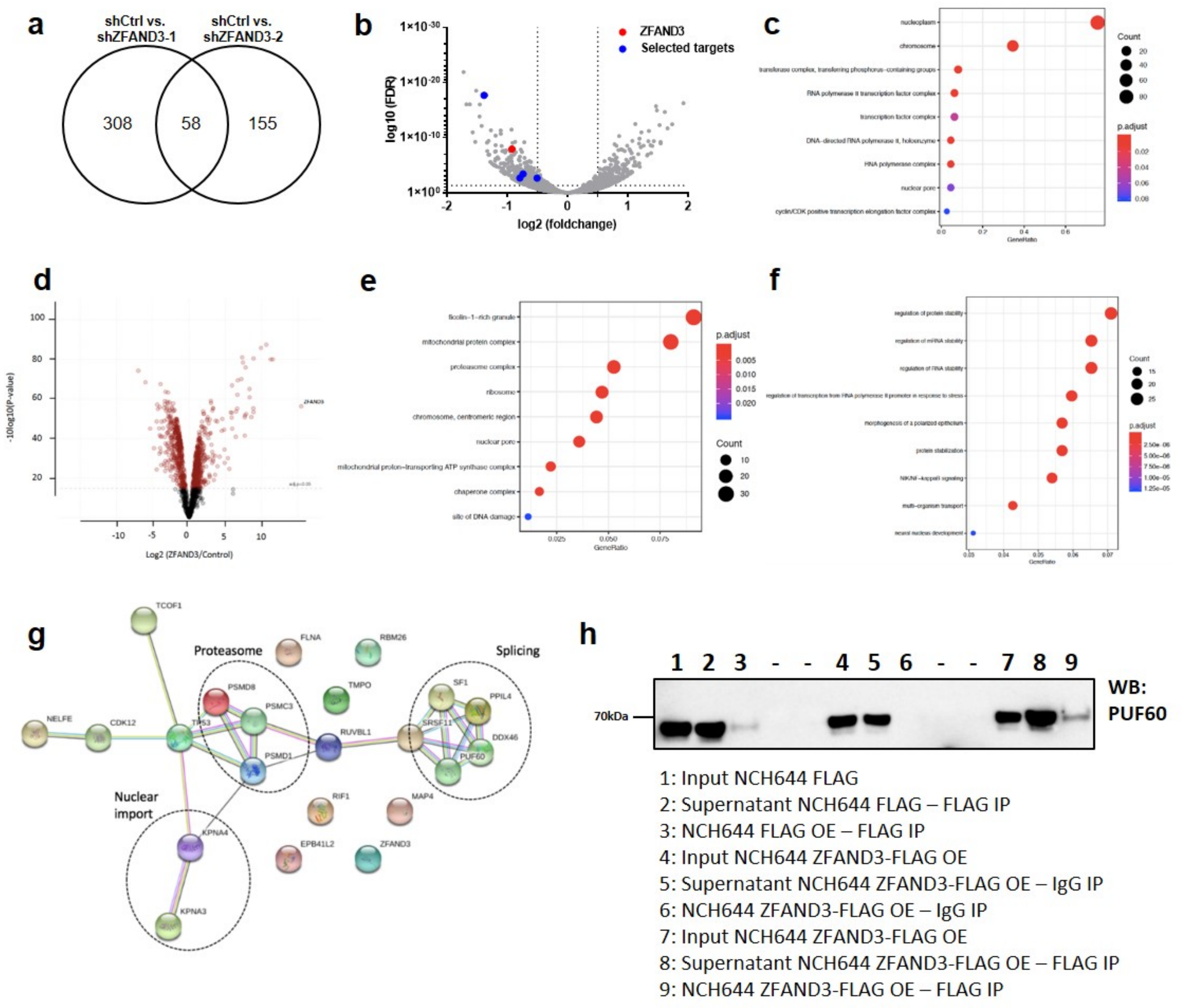

Supplementary Figure 7: Analysis of RNA sequencing, BioID and Co-IP/MS data. a) RNA sequencing was performed on control HI cells and two knockdown clones (shZFAND3-1 and shZFAND3-2). Differentially expressed genes were analysed at FDR=0.05, log2FC = -0.5 (n=3 per condition). Venn diagram displaying downregulated genes upon ZFAND3 KD resulting in 58 common genes in both shZFAND3-1 and shZFAND3-2. b) Volcano plot of RNA sequencing data of shCtrl compared to shZFAND3-1 HI cells. c) BioID analysis was performed in control NI and ZFAND3-FLAG overexpressing NI cells (n=3). GO analysis of the BioID results reveals enrichment of specific GO terms classified by cellular compartment. d) Volcano plot of Co-IP/MS results showing 432 proteins significantly enriched in ZFAND3-FLAG expressing cells compared to cells expressing FLAG-Tag alone. Two-sample t test was performed with a Benjamini-Hodgberg based FDR < 0.01. GO analysis reveals enrichment of specific GO terms classified by cellular compartment (e) or biological process (f) For the GO analysis p-value and q-value cutoff was at 5%, n and minimum 3 proteins per category as threshold. g) Interaction between 22 common proteins found in ZFAND3-FLAG IP and BioID were

determined by network analysis using STRING database. Splicing-related proteins, proteasome related proteins and nuclear import proteins are highlighted. h) Co-IP/Western blot from NI GSCs overexpressing either FLAG-tag alone (lanes 1-3) or FLAG-tagged ZFAND3 (lanes 4-9) demonstrating physiologically relevant protein-protein interaction between ZFAND3 and PUF60. FLAG-antibody was applied for lanes 1-3 and 7-9, while control IgG antibody was applied to lanes 4-6 (n=3).

Supplementary Figure 8

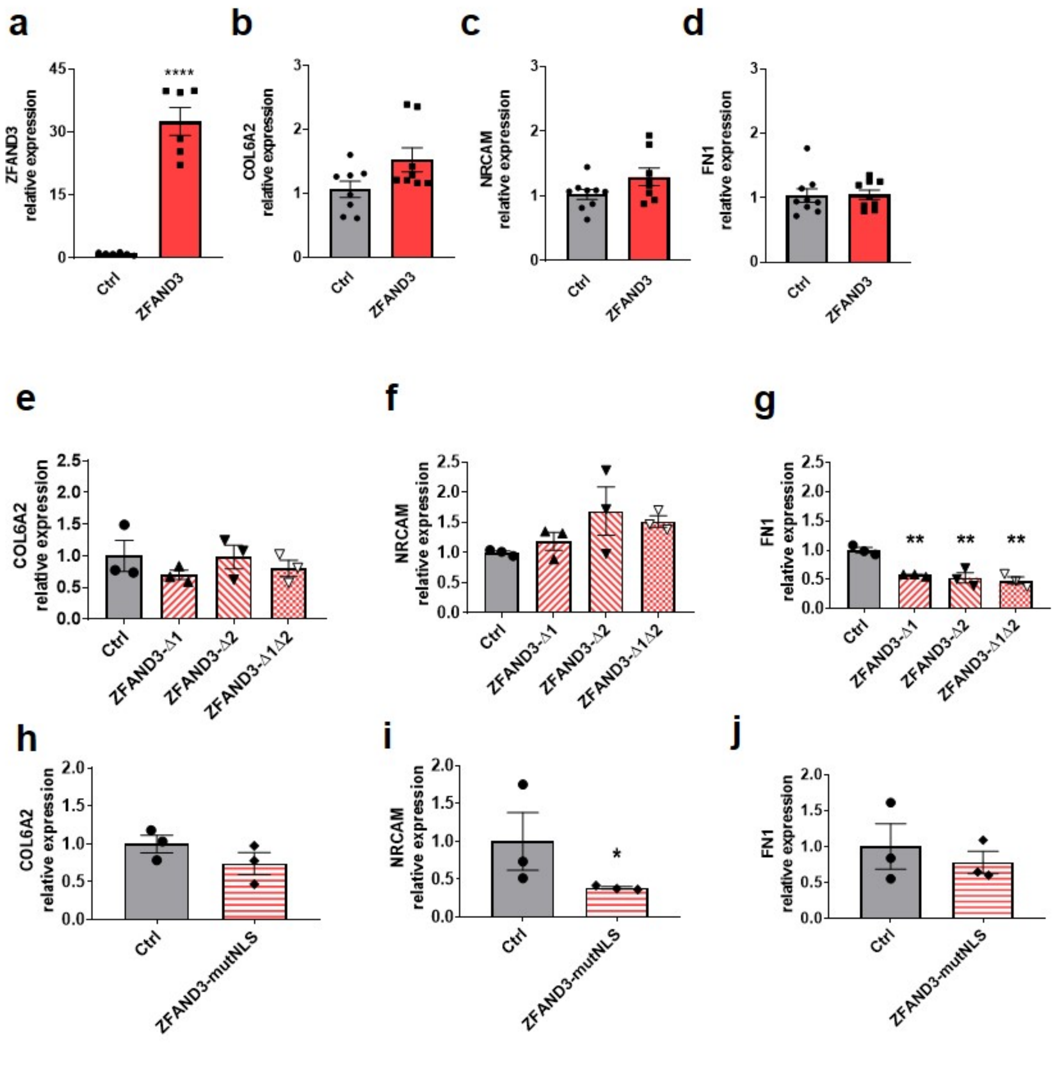

Supplementary Figure 8: Target gene expression upon stable overexpression of ZFAND3 and mutant constructs. qPCRs showing relative expression of *ZFAND3* (n=6) (a), *COL6A2* (n=8) (b), *NRCAM* (n=9) (c) and *FN1* (n=9) (d) in HI cells upon ZFAND3 overexpression. No increase in gene expression was observed upon overexpression of mutant constructs ZFAND3-Δ1, ZFAND3-Δ2, ZFAND3-Δ1Δ2, or ZFAND3-mutNLS (n=3). Results are displayed as average +/- SEM and results were analysed with an unpaired, two-sided t-test. $^{*}p_{value}<0.05$, $^{**}p_{value}<0.01$, $^{****}p_{value}<0.0001$.

Supplementary Figure 9

Figure 3b

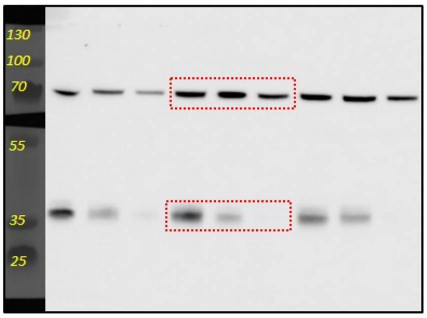

Figure 4b

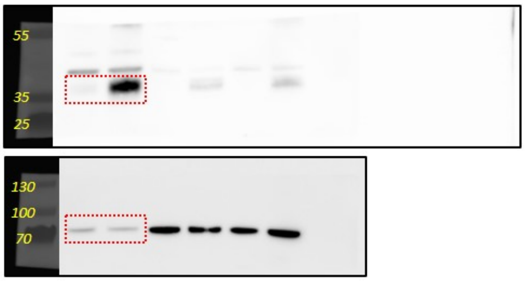

Figure 5c

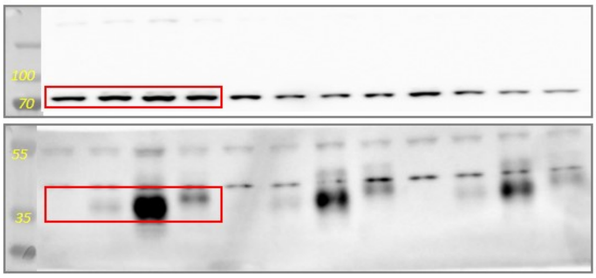

Figure 5i

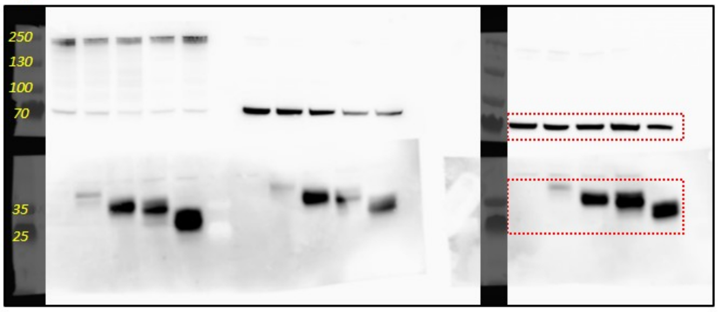

Figure S4b

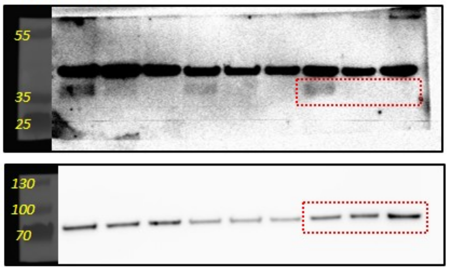

Figure S5c

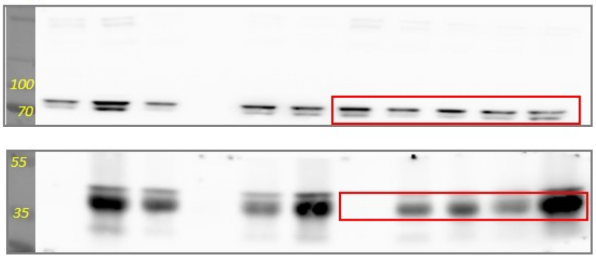

Figure S7h

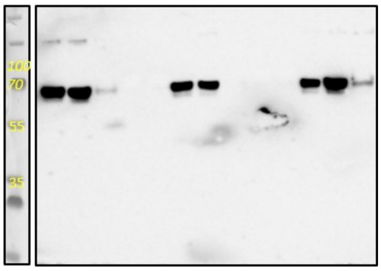

Supplementary Figure 9: Original Western blot membranes corresponding to indicated figures.

Supplementary Tables

Supplementary Table 1: Primer sequences

Gene	Forward primer	Reverse primer
CDH1 (N-cadherin)	GGTGGAGGAGAAGAAGACC	GGCATCAGGCTCCACAGT
COL6A2	GACTCCACCGAGATCGACCA	CTTGTAGCACTCTCCGTAGGC
EF1α	TTGTCGTCATTGGACACGTAG	TGCCACCGCATTTATAGATCAG
EGFR	CCCACTCATGCTCTACAACCC	TCGCACTTCTTACACTTGCGG
FN1	AGGAGCACCACCCCAGACATTACT	CCAGGCCGGGACTCAGGTTAT
MMP2	CGTCTGTCCCAGGATGACATC	ATGTCAGGAGAGGCCCCATA
NRCAM	TCCAACCATCACCCAACAGTC	TGAGTCCCATTACGGGTCCAG
NRP1	ACGTGGAAGTCTTCGATGGAG	CACCATGTGTTTCGTAGTCAGA
SNAI2	CGCCTCCAAAAAGCCAAACT	ACAGTGATGGGGCTGTATGC
ZEB1	TTCACAATTACTCACCTGTCCA	TGCGTCACATGTCTTTGATCTC
ZFAND3	CCAGACGATGATTCCGCTCC	GCGTGGTTATCGAGGTATTGTT
ZFAND3 (2)	CGATAACCACGCCAACTCTT	GGACCGTTCCGTATTCTCAA
COL6A2 S1 ChIP	TGAGCAAGCCGGACACAGGACA	TGGGACTCGCCCCTTGGTA
COL6A2 S2/3 ChIP	CAGCCAGGTCGTCCGGGAAAT	AGGCTGGAGGAGGTGGAGAG
COL6A2 S7 ChIP	TCGGGAGCGGAGCCTCCTCGGGA	AGGCTGGAGGAGGTGGAGAG
COL6A S8/9/10 ChIP1	GCGACTTGGGGCCACCTCCC	GACCCGAACCGCTCGCAGAC
COL6A2 S8/9/10 ChIP2	CCTGTGGCTCCGCGTCTCTG	GTGCCGGGGTCGTCTCGGGAG
NRCAM S1/2/3 ChIP	CTAAATCCCAGCCATCCTAGCC	CAACCTCCGTCAGCCTGCGA
NRCAM S4/5/6/7/8 ChIP1	AGCCCATTCGCTGCCGTC	CTCCCGCCCTCTCCGCTC
NRCAM S4/5/6/7/8 ChIP2	GTTGGCCAGGAGGGGAGGAG	ACCCTGGCGAAGCGAGGC
FN1 S7 ChIP1	GACCCCTAAGCATGTTGAGAC	GAAGGGATTTTTCCCGCAGGTT
FN1 S7 ChIP2	GACCCCTAAGCATGTTGAGAC	CACCTTCTTGGAGGCGACAAC

Supplementary Table 2: shRNA sequences against ZFAND3

Name	Target	Sequence	Company	Clone ID	Cataloge number
shZFAND3-1	ZFAND3	TACAGAACACATAACCGCA	Dharmacon	V3LHS_363696	RHS4531_EG60685
shZFAND3-2	ZFAND3	TAGTGAACTAAGAACAGCG		V3LHS_409051	

Supplementary Table 3: Antibodies

Target	Company	Catalog number	Purpose and dilution
HA	Sigma	H6908	WB 1:500 ; IF 1:100
mouse IgG1	Abcam	ab18447	Co-IP 7µg/mg beads

mouse IgG1 isotype control	CST	5415S	Co-IP 5µg/mg beads
FLAG M2	Sigma	F1804	Co-IP 5 µg/mg beads, WB 1:1000
Lamin B1	Abcam	ab16048	WB 1:1000
P53	Ventana Medical Systems, Roche	790-2912	clone: DO7, ready-to-use
Vimentin	Millipore	MAB3400	IHC 1:200
ZFAND3	Atlas Antibodies	HPA016755	WB: 1:250 - 1:500; IF culture: 1:100; IHC 1:50
ZFAND3	Atlas Antibodies	HPA016755	IHC: 1:600, IF: 1:1000 (paraffin sections)
GAPDH	Cell Signaling	5174S	WB 1:10000
COL VI	Abcam	ab182744	WB 1:000
Fibronectin 1	Abcam	ab2413	WB 1:500-1000
NRCAM	Cell Signaling	55284	WB 1:500
ZBED4	Bio-Connect	orb158740	WB: 1:2000 ChIP
PUF60/Pontin52	Atlas Antibodies	HPA059714	WB: 1:500
TIP49/RUVBL1	abcam	ab51500	WB: 1:100
TCOF1/Treacle	Atlas Antibodies	HPA038237	WB: 1:500
Iba1	Wako Pure Chemical Industries		IF: 1:3000
NeuN	Chemicon	clone: A60	IF: 1:500
Goat anti Mouse IgG HRP	Jackson Laboratory	115-035-003	WB 1:10000
Goat anti Rabbit HRP	Jackson Laboratory	111-035-003	WB 1:00000
Goat anti Rabbit IgG Alexa 555	Invitrogen	A21428	IF: 1:1000

Supplementary Table 4: Constructs

Name used in publication	***Full Plasmid name***	***Purpose***	***Origin***	***Cell line***
shScr	pGIPZ-sh non targeting control	Control, non targeting sh expressing GFP	Open Biosystems/GE Dharmacon	HI (NCH601), HI-2 (NCH465)
shZFAND3-1	pGIPZ-shZFAND3-1	ZFAND3 knockdown mature antisense: TACAGAACACATAACCGCA expressing GFP	Open Biosystems/GE Dharmacon (V3LHS_363696)	HI (NCH601), HI-2 (NCH465)
shZFAND3-2	pGIPz-shZFAND3-2	ZFAND3 knowckdown mature antisense: TAGTGAACTAAGAACAGCG expressing GFP	Open Biosystems/GE Dharmacon (V3LHS_409051)	HI (NCH601), HI-2 (NCH465)
Ex-Ctrl	Ex-negcon. LV225	lentiviral plasmid; EF1promoter-IRES2-eGFP-IRES-puromycin	GeneCopoeia	NI (NCH644)
Ex-ZFAND3	Ex-ZFAND3-LV225	lentiviral plasmid; EF1promoter-ZFAND3-IRES2-eGFP-IRES-puromycin	GeneCopoeia	NI (NCH644)
NA	pCDH-EF1α-MCS-IRES-Neo	basic lentiviral plasmid for all this paper overexpression plasmids	System Bioscience (#CD533A-2)	NA
Ctrl-HA	pCDH-EF1-HA-IRES-neo	HA-tag only control plasmid	this paper	NI (NCH644)

ZFAND3-OE	pCDH-EF1-ZFAND3-HA-neo	ZFAND3 overexpression with C-terminal HA-tag	this paper	NI (NCH644)
ZFAND3 Δ1	pCDH-EF1-ZFAND3_DD1-HA-IRES-neo	ZFAND3 with deletion of Znfinger domain1, expression as HA-tagged protein	this paper	NI (NCH644)
ZFAND3 Δ2	pCDH-EF1-ZFAND3_DD2-HA-IRES-neo	ZFAND3 with deletion of Znfinger domain2, expression as HA-tagged protein	this paper	NI (NCH644)
ZFAND3 Δ1Δ2	pCDHEF1-ZFAND3_DD1D2-HA-IRES-neo	ZFAND3 with deletion of both Znfinger domains, expression as HA-tagged protein	this paper	NI (NCH644)
ZFAND3-OE-mutNLS	pCDH EF1-ZFAND3mutNLS-HA -IRES-neo	overexpression of ZFAND3 with mutated NLS and HA tag	this paper	NI (NCH644)
ZFAND3-OE-mutNLS-NLS	pCDH EF1-ZFAND3mutNLS-NLS-HA -IRES-neo	overexpression of ZFAND3 with mutated NLS fused to cMYC NLS (PAAKRVKLDG) and HA tag	this paper	NI (NCH644)
NA	pcDNA3.1 myc BioID	non lentiviral plasmid overexpressiong myc-BirA(R118G)	gift from Kyle Roux (Addgene plasmid # 35700 ; http://n2t.net/addgene:35700 ; RRID:Addgene_35700)	NA
myc-BioID	pCDH-EF1-myc-BioID-IRES-neo	overexpression of BioID with N-terminal myc-tag	this paper (myc_BioID was amplified from Addgene plasmid #35700)	NI (NCH644)
myc-BioID-ZFAND3	pCDH EF1-myc-BioID-ZFAND3-IRES-neo	overexpression of myc_BioID-ZFAND3 fusion protein	this paper	NI (NCH644)
Ctrl-Flag	pCDH-EF1-3xflag-IRES-neo	Overexpression of 3x Flag tag	This paper	NI (NCH644)
ZFAND3-OE Flag	pCDH-EF1-ZFAND3-3xflag-IRES-neo	overexpression of ZFAND3 with a C-terminal 3xflag tag	this paper	NI (NCH644)
ZFAND3_M1	pCDH-EF1-ZFAND3_M1-3xflag-IRES-neo	overexpression of mutated ZFAND3 (C32A + C35A) with a C-terminal 3xflag tag	this paper	NI (NCH644)
ZFAND3_M2	pCDH-EF1-ZFAND3_M2-3xflag-IRES-neo	overexpression of mutated ZFAND3 (C176A +C181A)with a C-terminal 3xflag tag	this paper	NI (NCH644)
ZFAND3_M1M2	pCDH-EF1-ZFAND3_M1M2-3xflag-IRES-neo	overexpression of mutated ZFAND3 (C32A + C35A + C176A +C181A)with a C-terminal 3xflag tag	this paper	NI (NCH644)

Supplementary Table 5: Promotor sequences (for Reporter Assay)

<u>COL6A2</u>
CCTGACCCGGGGCCTCTCGCGGGAGGCCTGAGCAAGCCGGACACAGGACACGGGGTAGGGGAGGGGTCGGGGGGCTGATGGG
GGGAACCCTGCACCCCCCAGGGCAGCTGCTACCAAGGGGCGAGTCCCAGGGCCCCCGTCGGCCCTGCGTGCGGGGCGCGGTCCC
CAACACCCAGGGCCCCGGAGGCGGACACAGCCCCAGCCAGGTCGTCCGGGAAATGGGGCGGGGGCGACGGGCGG
CCGGGCCCGGGACGCGAAGTCCGAGCAGCAGCGGGCAGGGGCTGGCGGGGGAGCTCGGCC
CGGGCTGCAGGGGGGTCCCCACCCTCTCCACCTCCTCCAGCCTCCCGCCCTCGAGGGTCC
CCGCTTCCCTCCCATCCCCCTCCCGTGCCCCCGGCCCCCTCCTCCCATCCGCGGGGCCGC
AGCGCTTCCTGGCGGCGGGGCGGGTCAGGCCGGCGGGGCGGGGTATAAAGGGGGCGGCGC
CGGCCGCGGTTCCCTCCCTGCTGCTTACTCGGCGCCCGCGCCTCGGGCCGTCGGGAGCGG
AGCCTCCTCGGGACCAGGTGAGCGCCTCCCGGACCCCGCACCCTGGAAGCCGCTCGGCCC
GCGGGGGGTGACCCCGAGTCCTGGGAAGGCGGCGGCGGCGGCTCCGTCCCTCGGGTCCCC
GGGAAGGGGGACTCCAGCCCCAGGGACGGCGGGGGGCTCGGCGGGTTCGGGGCTCCTCCT
CGCGGGGCTGGGGCCGCGCCTGCCCCTGTGGCTCCGCGTCTCTGGGTCCGACCCTCGGGC
GCGCGACTTGGGGCCACCTCCCCGCGGCCTCCTCTGGGGCGGAGCCGGCCTGGGCGGGGT
GGGGGGGTCCCTGTCTGCGCCCGAGCTCGGTGCTGGGACCCCCGCTCCCGAGACGACCCC
GGCACCGCACGCCCCGCCAGGCCCCGCGTCTGCGAGCGGTTCGGGTCCGGCTCCGGCCCG
CGGGGAAGACGCCCCGGCTGGCTGGGACCTCCGGGGGCGCAGGGCCTCTCCCCGGGCCGG
ACGGAAGGGGCGGCGGGGCGGGGGGAGGAGGGGCTTTCGGTGCCCGAGGGCGGGACTGGG
CGGGGAGGGGACGCGGGTGGCCCCGACGCCCCATCGCTGCGCCCCTCCCGGCCTGGAGCC

<u>COL6A2mut</u>
CCTGACCCGGGGCCTCTCGCGGGAGGCCTGAGCAAGCCGGACACAGGACACGGGGTAGGG
GAGGGGTCGGGGGGCTGATGGGGGGAACCCTGCACCCACCAGGGCAGCTGCTACCAAGGG
GCGAGTCCCAGGGCCCCCGTCGGCCCTGCGTGCGGGGCGCGGTCCCCAACACCCAGGGCC
CCGGAGGCGGACACAGCCCCAGCCAGGTCGTCCGGGAAATGGGGCGATGGCGACGGGCGG
CCGGGCCCGGGACGCGAAGTCCGAGCAGCAGCGGGCAGGGGCTGGCGGTGGAGCTCGGCC
CGGGCTGCAGGGGGGTCCCCACCCTCTCCACCTCCTCCAGCCTCCCGCTCTCGAGGGTCC
CCGCTTCCCTCCCATCCCCCTCCCGTGCCCCCGGCCCCCTCCTCCCATCCGCGGGGCCGC
AGCGCTTCCTGGCGGCGGGGCGGGTCAGGCCGGCGGCGCGGGGTATAAAGGTGGCGGCGC
CGGCCGCGGTTCCCTCCCTGCTGCTTACTCGGCGCCCGCGCCTCGGGCCGTCGGGAGCGG
AGCCTCCTCGGGACCAGGTGAGCGCCTCCCGGACCCCGCACCCTGGAAGCCGCTCGGCCC
GCGGGTGGTGACCCCGAGTCCTGGGAAGGCGGCGGCGGCGGCTCCGTCCCTCGGGTCCCC
GGGAAGGGGGACTCCAGCCCCAGGGACGGCGGGGGGCTCGGCGGGTTCGGGGCTCCTCCT
CGCGGGGCTGGGGCCGCGCCTGCCCCTGTGGCTCCGCGTCTCTGGGTCCGACCCTCGGGC
GCGCGACTTGGGGCCACCTCCCCGCGGCCTCCTCTGGTGCGGAGCCGGCCTGTGCGGGGT
GTGGGGGTCCCTGTCTGCGCCCGAGCTCGGTGCTGGGACCCCCGCTCCCGAGACGACCCC
GGCACCGCACGCCCCGCCAGGCCCCGCGTCTGCGAGCGGTTCGGGTCCGGCTCCGGCCCG
CGGGGAAGACGCCCCGGCTGGCTGGGACCTCCGGGGGCGCAGGGCCTCTCCCCGGGCCGG
ACGGAAGGCGCGGCGGGGCGGGTGGAGGAGGGGCTTTCGGTGCCCGAGCGCGGGACTGGG
CGGGGAGGGGACGCGGGTGGCCCCGACGCCCCATCGCTGCGCCCCTCCCGGCCTGGAGCC

<u>NRCAM</u>
AAAACAAAACCCCCAAAACCACTCACCCAATCTCAACCACCCCAACTGATGAATTTCGAA
GAAAGTGCTTTTTAAAAAAGAAAAAACTAGAAGACCACAGACCTCCCACAGAAAGCTAAA
CCCATGAGAGTGGCATTCAAAAACTACCCATTTTCTGGAACATCTTTTGTAGCTAAATCC
CAGCCATCCTAGCCCCGGAACACTCCCGCCCTCCGTCCCCGCACCCCCACCCCCGCCGCC
ACACGCGCTCGCAGGCTGACGGAGGTTGGCGGTGGGGCGGCCGCTCAGGTGAGGGGCCAC
CCAGTCCCTCGAGGCGCCGGCGGCTGGAGCCCATTCGCTGCCGTCGCAGCAGCAGGGCAA
GAGGGGGTTGGCCAGGAGGGGAGGAGGCGGAGGACGCCCGCAGGGAGTGCGGGGGAGGGG
GACGGGAGGACGCTAAGGAGAGCCGGGAGGGGCAAAAGCACGGCGCGGGGAGGGGGAGCG
GAGAGGGCGGGAGCGGCGGGGGCGCGCGCCGGCGCGGGGCCAGCCTCGCTTCGCCAGGGT
CGCTGGCGGACGCGCGCCGCAGTCCGAGCCTCAGACGCGCGGGCGCGGGGGACGGCGCAG

<u>NRCAMmut</u>
AAAACAAAACCCCCAAAACCACTCACCCAATCTCAACCACCCCAACTGATGAATTTCGAA

GAAAGTGCTTTTTAAAAAAGAAAAAACTAGAAGACCACAGACCTCCCACAGAAAGCTAAA
CCCATGAGAGTGGCATTCAAAAACTACCCATTTTCTGGAACATCTTTTGTAGCTAAATCC
CAGCCATCCTAGCCCCGGAACACTCCCGCTCTCCGTCCCCGCACCCACACGCCCGCCGCC
ACACGCGCTCGCAGGCTGACGGAGGTTGGCGGTGGGGCGGCCGCTCAGGTGAGGGGCCAC
CCAGTCCCTCGAGGCGCCGGCGGCTGGAGCCCATTCGCTGCCGTCGCAGCAGCAGGGCAA
GAGGGGGTTGGCCAGGAGGGGAGGAGGCGGAGGACGCCCGCAGGGAGTGCGGCGGAGGCG
GACGGGAGGACGCTAAGGAGAGCCGGGAGGGGCAAAAGCACGGCGCGGGGAGGCGGAGCG
GAGAGCGCGGGAGCGGCGGTGGCGCGCGCCGGCGCGGGGCCAGCCTCGCTTCGCCAGGGT
CGCTGGCGGACGCGCGCCGCAGTCCGAGCCTCAGACGCGCGGGCGCGGGGGACGGCGCAG

<u>FN1</u>
TCCCTTCCCCCATCCCCTAAAAAGTTTGATGACCGCAAAGGAAACCGAAAAAAAGTTGTC
TTGCCCCAGTCCTGGCGGGCCATCAGCATCTCTTTTGTTCGCTGCGAACCCACAGTCCCC
CGTGACGTCACCCGGAGCCCGGGCCAATCGGCGCGCGGTCGGCTGCGGCGGCCGGCGGGC
GGGCGGGCGGGTGGGGTGGGGCGGGGCGGGGACAGCCCGGCGGGTCTCTCCTCCCCCGCG
CCCCGGGCCTCCAGAGGGGCGGGAGGGGACCGTCCCATATAAGCCCCGGCTCCCGGCGCT
CGGACGCCCGCGCCGGCTGTGCTGCACAGGGGGAGGAGAGGGAACCCCAGGCGCGAGCGG
GAAGAGGGGACCTGCAGCCACAACTTCTCTGGTCCTCTGCATCCCTTCTGTCCCTCCACC
CGTCCCCTTCCCCACCCTCTGGCCCCCACCTTCTTGGAGGCGACAACCCCCGGGAGGCAT
TAGAAGGGATTTTTCCCGCAGGTTGCGAAGGGAAGCAAACTTGGTGGCAACTTGCCTCCC
GGTGCGGGCGTCTCTCCCCCACCGTCTCAACATGCTTAGGGGTCCGGGGCCCGGGCTGCT

FN1_no ATG (used in HEK)
TCCCTTCCCCCATCCCCTAAAAAGTTTGATGACCGCAAAGGAAACCGAAAAAAAGTTGTCTTGCCCCAGTCCTGGCGGGCCATCAG
CATCTCTTTTGTTCGCTGCGAACCCACAGTCCCCCGTGACGTCACCCGGAGCCCGGGCCAATCGGCGCGCGGTCGGCTGCGGCGG
CCGGCGGGCGGGCGGGCGGGTGGGGTGGGGCGGGGCGGGGACAGCCCGGCGGGTCTCTCCTCCCCCGCGCCCCGGGCCTCCA
GAGGGGCGGGAGGGGACCGTCCCATATAAGCCCCGGCTCCCGGCGCTCGGACGCCCGCGCCGGCTGTGCTGCACAGGGGGAGG
AGAGGGAACCCCAGGCGCGAGCGGGAAGAGGGGACCTGCAGCCACAACTTCTCTGGTCCTCTGCATCCCTTCTGTCCCTCCACCC
GTCCCCTTCCCCACCCTCTGGCCCCCACCTTCTTGGAGGCGACAACCCCCGGGAGGCATTAGAAGGGATTTTTCCCGCAGGTTGCG
AAGGGAAGCAAACTTGGTGGCAACTTGCCTCCCGGTGCGGGCGTCTCTCCCCCACCGTCTCAAC

FN1mut_no ATG (used in HEK)
TCCCTTCGCCCATCCCCTAAAAAGTTTGATGACCGCAAAGGAAACCGAAAAAAAGTTGTCTTGCCCCAGTCCTGGCGGGCCATCAG
CATCTCTTTTGTTCGCTGCGAACCCACAGTCCCCCGTGACGTCACCCGGAGCCCGGGCCAATCGGCGCGCGGTCGGCTGCGGCGG
CCGGCGGGCGGGCGGACGGGTGGGGTGAGGCGGGGCCGGGACAGCCCGGCGGGTCTCTCCTCGCCCGCGCCCCGGGCCTCCAG
AGGAGCGGGAGGGGACCGTCCCATATAAGCCCCGGCTCCCGGCGCTCGGACGCCCGCGCCGGCTGTGCTGCACAGGGGGAGGA
GAGGGAACCCCAGGCGCGAGCGGGAAGAGGGGACCTGCAGCCACAACTTCTCTGGTCCTCTGCATCCCTTCTGTCCCTCCACCCG
TCCCCTTCCCCACCCTCTGGCCCCCACCTTCTTGGAGGCGACAACCCCCGGGAGGCATTAGAAGGGATTTTTCCCGCAGGTTGCGA
AGGGAAGCAAACTTGGTGGCAACTTGCCTCCCGGTGCGGGCGTCTCT

Supplementary Table 9: Anonymized patient demographics

ID	*Sexe*	*Age diagnosis*
1	Male	83
2	Male	43
3	Male	80
4	Male	71
5	Male	65
6	Female	60
7	Male	82
8	Male	52
9	Female	38
10	Female	75
11	Male	80
12	Male	57
13	Male	57
14	Female	35
15	Male	75
16	Female	85
17	Female	72

Supplementary References

1. Bowman, R.L., Wang, Q., Carro, A., Verhaak, R.G. & Squatrito, M. GlioVis data portal for visualization and analysis of brain tumor expression datasets. *Neuro Oncol* **19**, 139-141 (2017).

2. Tang, Z. *et al.* GEPIA: a web server for cancer and normal gene expression profiling and interactive analyses. *Nucleic Acids Res* **45**, W98-W102 (2017).
