# Supplementary material for: AN1-type zinc finger protein 3 (ZFAND3) is a transcriptional regulator that drives Glioblastoma invasion
Source: Nat Commun. 2020 Dec 11;11:6366. doi: 10.1038/s41467-020-20029-y (PMC7732990; doi:10.1038/s41467-020-20029-y)
Supplement: Supplementary file 3 — Reporting Summary [file 41467_2020_20029_MOESM3_ESM.pdf]

nature research

Corresponding author(s): Simone P. Niclou
Last updated by author(s): Oct 12, 2020

Reporting Summary

Nature Research wishes to improve the reproducibility of the work that we publish. This form provides structure for consistency and transparency in reporting. For further information on Nature Research policies, see our Editorial Policies and the Editorial Policy Checklist.

Statistics

For all statistical analyses, confirm that the following items are present in the figure legend, table legend, main text, or Methods section.

n/a	Confirmed	
☐	☒	The exact sample size (*n*) for each experimental group/condition, given as a discrete number and unit of measurement
☐	☒	A statement on whether measurements were taken from distinct samples or whether the same sample was measured repeatedly
☐	☒	The statistical test(s) used AND whether they are one- or two-sided<br>*Only common tests should be described solely by name; describe more complex techniques in the Methods section.*
☒	☐	A description of all covariates tested
☒	☐	A description of any assumptions or corrections, such as tests of normality and adjustment for multiple comparisons
☐	☒	A full description of the statistical parameters including central tendency (e.g. means) or other basic estimates (e.g. regression coefficient) AND variation (e.g. standard deviation) or associated estimates of uncertainty (e.g. confidence intervals)
☐	☒	For null hypothesis testing, the test statistic (e.g. F, t, r) with confidence intervals, effect sizes, degrees of freedom and P value noted<br>*Give P values as exact values whenever suitable.*
☒	☐	For Bayesian analysis, information on the choice of priors and Markov chain Monte Carlo settings
☒	☐	For hierarchical and complex designs, identification of the appropriate level for tests and full reporting of outcomes
☒	☐	Estimates of effect sizes (e.g. Cohen's *d*, Pearson's *r*), indicating how they were calculated

Our web collection on statistics for biologists contains articles on many of the points above.

Software and code

Policy information about availability of computer code

Data collection	For data collection we have used the following softwares, which are all mentioned in the Method section:<br>Ni-E microscope (Nikon), Incucyte Zoom, Evos cell imaging system, Zeiss Zen software, QuantStudioTM 5, ImageQuant LAS4010 imaging station and software, LC-MS/MS on a Q-Exactive Plus mass spectrometer (Thermo) connected to a Dionex Ultimate 3000 (Thermo), run in trap mode using a Acclaim Pepmap 100 trap column (Dionex), ClarioStar plate reader, NextSeq 500 (Illumina), Leica DM6000B microscope, Olympus DP72 camera.
Data analysis	For data analysis we have used the following softwares:<br>QuantStudio software 7, MaxQuant software package 1.6.5.0, Incucyte Zoom 2018A, Graph Pad Prism 8, Image J 1.52a., Zeiss Zen software 2.3, Microsoft EXCEL 2013 (15.0.5275.1000), Bowtie (v2-2.3.2), Visiopharm software V6.6.1, R-software 3.4.1

For manuscripts utilizing custom algorithms or software that are central to the research but not yet described in published literature, software must be made available to editors and reviewers. We strongly encourage code deposition in a community repository (e.g. GitHub). See the Nature Research guidelines for submitting code & software for further information.

Data

Policy information about availability of data

All manuscripts must include a data availability statement. This statement should provide the following information, where applicable:

- Accession codes, unique identifiers, or web links for publicly available datasets
- A list of figures that have associated raw data
- A description of any restrictions on data availability

A data availability statement is included in the manuscript. The datasets generated and analyzed during the current study are available in the GEO repository (accession number GSE138618) for RNA sequencing, in the GEO repository (accession number GSE134470) for DNA microarrays and in the The MassIVE site repository (University of California San Diego, ProteomeXchange consortia) under (ftp://massive.ucsd.edu/MSV000086247/) for raw proteomics data.

Field-specific reporting

Please select the one below that is the best fit for your research. If you are not sure, read the appropriate sections before making your selection.

[x] Life sciences [ ] Behavioural & social sciences [ ] Ecological, evolutionary & environmental sciences

For a reference copy of the document with all sections, see nature.com/documents/nr-reporting-summary-flat.pdf

Life sciences study design

All studies must disclose on these points even when the disclosure is negative.

Sample size	The number of replicates for in vitro bioassays was based on previous experience. The minimal sample size was generally 3 biological replicates, an increased sample size was preferred whenever possible. No sample size calculation was performed for in vivo experiments.
Data exclusions	No data were excluded from the analyses. All cells/animals that met proper technical and experimental conditions were included in the analysis.
Replication	One graph from the original submission (induction of gene expression of target genes in ZFAND3 overexpressing cells) could not be reproduced. The new data (with multiple replicates) has been added and is now presented in suppl. figure 8. All other data has been reproduced multiple times. For all other data, the experiments were replicated in at least 2 biological independent experiments.
Randomization	No randomization was done to allocate experimental groups in vivo, randomization was not applicable due to the implanted constructs.
Blinding	In vivo experiments were blinded to group allocation during data collection and analysis.

Reporting for specific materials, systems and methods

We require information from authors about some types of materials, experimental systems and methods used in many studies. Here, indicate whether each material, system or method listed is relevant to your study. If you are not sure if a list item applies to your research, read the appropriate section before selecting a response.

Materials & experimental systems

n/a	Involved in the study	
[ ]	[x]	Antibodies
[ ]	[x]	Eukaryotic cell lines
[x]	[ ]	Palaeontology and archaeology
[ ]	[x]	Animals and other organisms
[ ]	[x]	Human research participants
[x]	[ ]	Clinical data
[x]	[ ]	Dual use research of concern

Methods

n/a	Involved in the study	
[x]	[ ]	ChIP-seq
[x]	[ ]	Flow cytometry
[x]	[ ]	MRI-based neuroimaging

Antibodies

Antibodies used	A list of all antibodies used in the study is provided in Suppl. Table 3.
Validation	All primary antibodies were used according to the manufacturer's instructions for Western Blotting, IHC, IF and co-IP. Details of experimental conditions are provided in Suppl. Table 3.

Eukaryotic cell lines

Policy information about cell lines

Cell line source(s)	The patient-derived GBM serum-free cultures were provided by Prof. Christel Herold-Mende (Department of Neurosurgery, University of Heidelberg, Germany) for NCH601, NCH465, NCH644 and NCH660h lines, or by Prof. Rolf Bjerkvig (University of Bergen, Norway) for GG6 and BG7 lines. HEK293T and U87 cells were obtained from ATCC (HTB-14).
Authentication	Authentication (to original patient) was based on methylation profiling and expression arrays.
Mycoplasma contamination	All of the cell lines were tested regularly for mycoplasma contamination and only mycoplasma negative cells were used in this study.
Commonly misidentified lines (See ICLAC register)	None of the cell lines used are listed in the ICLAC register.

Animals and other organisms

Policy information about studies involving animals; ARRIVE guidelines recommended for reporting animal research

Laboratory animals	NOD/SCID mice (Charles River), at least 5 weeks of age, m/f. Animals were housed in individually ventilated cages in a Specific Pathogen Free (SPF) Facility, under controlled environment (temperature set at 22+/-2°C , humidity was kept between 45% and 65% and dark/light cycle is 12h/12h) with free access to water and food ad libitum.
Wild animals	The study did not involve wild animals.
Field-collected samples	The study did not involve samples collected from the field.
Ethics oversight	Study protocol for animal experimentation was approved by the responsible authorities in Luxembourg from Ministry of Health and Ministry of Agriculture (LUPA2017/15) and the local ethical committee called Animal Welfare Structure of the Luxembourg Institute of Health approved the protocol.

Note that full information on the approval of the study protocol must also be provided in the manuscript.

Human research participants

Policy information about studies involving human research participants

Population characteristics	The 17 included patients were all diagnosed with Glioblastoma according to the WHO2016 classification, and comprised 11 males and 6 females with a mean age at diagnosis of 65.29 years (range 35-85 years). Population characteristics of the human research participants are listed in detail in table S9.
Recruitment	Archived formaldehyde-fixed-paraffin-embedded tissue samples from all consecutive glioblastoma patients diagnosed between 2010-2014 at Odense University Hospital, Odense, Denmark, were screened for P53 positivity and simultaneous inclusion of areas with diffuse tumor infiltration, as described in M&M. All patients that fulfilled these criteria, were included in the final patient cohort.
Ethics oversight	Approved by the Danish Data Inspection Authority (approval number 16/11065) and the Regional Scientific Ethical Committee of the Region of Southern Denmark (approval number S-20150148).

Note that full information on the approval of the study protocol must also be provided in the manuscript.
